# Metformin Use Is Associated With Reduced Mortality in a Diverse Population With COVID-19 and Diabetes

**DOI:** 10.3389/fendo.2020.600439

**Published:** 2021-01-13

**Authors:** Andrew B. Crouse, Tiffany Grimes, Peng Li, Matthew Might, Fernando Ovalle, Anath Shalev

**Affiliations:** ^1^Hugh Kaul Precision Medicine Institute, University of Alabama at Birmingham, Birmingham, AL, United States; ^2^Comprehensive Diabetes Center, Department of Medicine, Division of Endocrinology, Diabetes and Metabolism, University of Alabama at Birmingham, Birmingham, AL, United States; ^3^School of Nursing, University of Alabama at Birmingham, Birmingham, AL, United States

**Keywords:** African-American, coronavirus disease-2019, diabetes, metformin, mortality

## Abstract

**Background:**

Coronavirus disease-2019 (COVID-19) is a growing pandemic with an increasing death toll that has been linked to various comorbidities as well as racial disparity. However, the specific characteristics of these at-risk populations are still not known and approaches to lower mortality are lacking.

**Methods:**

We conducted a retrospective electronic health record data analysis of 25,326 subjects tested for COVID-19 between 2/25/20 and 6/22/20 at the University of Alabama at Birmingham Hospital, a tertiary health care center in the racially diverse Southern U.S. The primary outcome was mortality in COVID-19-positive subjects and the association with subject characteristics and comorbidities was analyzed using simple and multiple linear logistic regression.

**Results:**

The odds ratio of contracting COVID-19 was disproportionately high in Blacks/African-Americans (OR 2.6; 95% CI 2.19–3.10; p<0.0001) and in subjects with obesity (OR 1.93; 95% CI 1.64–2.28; p<0.0001), hypertension (OR 2.46; 95% CI 2.07–2.93; p<0.0001), and diabetes (OR 2.11; 95% CI 1.78–2.48; p<0.0001). Diabetes was also associated with a dramatic increase in mortality (OR 3.62; 95% CI 2.11–6.2; p<0.0001) and emerged as an independent risk factor in this diverse population even after correcting for age, race, sex, obesity, and hypertension. Interestingly, we found that metformin treatment prior to diagnosis of COVID-19 was independently associated with a significant reduction in mortality in subjects with diabetes and COVID-19 (OR 0.33; 95% CI 0.13–0.84; p=0.0210).

**Conclusion:**

Thus, these results suggest that while diabetes is an independent risk factor for COVID-19-related mortality, this risk is dramatically reduced in subjects taking metformin prior to diagnosis of COVID-19, raising the possibility that metformin may provide a protective approach in this high risk population.

## Introduction

Coronavirus disease-2019 (COVID-19) caused by the severe acute respiratory syndrome-coronavirus-2 (SARS-CoV-2) is a growing global pandemic that has devastated Asia, Europe, and now the United States. Its increasing death toll has been linked to higher age and a number of comorbidities including hypertension, obesity, and diabetes (1, 2), but approaches to counteract this trend are still lacking. Being a new disease, the specific patient characteristics of these at risk populations are also only starting to emerge with studies reported from China (3–5), Europe (6, 7) and more recently New York (1, 2).

However, currently still very little is known about patient characteristics in the U.S., particularly in more diverse communities with a large proportion of Blacks/African-Americans such as in the South. This information is especially relevant as African-Americans have been disproportionally affected by this pandemic across the nation (8–10) and the prevalence of comorbidities including diabetes is very high in these communities (11). We therefore conducted a retrospective observational study of subjects diagnosed with COVID-19 at the University of Alabama at Birmingham (UAB) Hospital, a tertiary health care center in the South, aimed at identifying the patient characteristics and factors affecting mortality especially in the context of diabetes in this diverse cohort.

## Methods

### Study Design and Participants

We conducted a retrospective analysis of de-identified electronic health record data (EHR). The sampling method consisted of including subjects consecutively tested for COVID-19 between February 25, 2020 and June 22, 2020 at UAB (Institutional Review Board protocol E160105006). To make the results as generalizable as possible and minimize any selection bias, completed testing within that time frame was also the only inclusion criteria and lack of outcome data in terms of survival was the only exclusion criteria. Subjects were categorized as confirmed COVID-19 positive or negative based on RT-PCR results from SARS-CoV-2 viral nucleic acid testing in respiratory specimens. The primary outcome was mortality and the effects of patient characteristics and comorbidities as documented in the EHR data (including 12 months before COVID-19 diagnosis) were analyzed. EHR data definitions for obesity included a body mass index (BMI) of ≥30 kg/m^2^ and for hypertension a systolic blood pressure of ≥140 mmHg and/or a diastolic blood pressure of ≥90 mmHg. HbA1C was analyzed as a continuous variable. In terms of treatment, we focused on metformin and insulin as they were the two most common used drugs for diabetes and reliable electronic health record data were available. The number of subjects on other antidiabetic medications such as sodium-glucose cotransporter 2 (SGLT2) inhibitors or dipeptidyl peptidase IV (DPPIV) inhibitors was too small to allow for meaningful statistical analysis. This may have been due to the much higher costs of these newer medications and our cohort that included underserved communities.

### Statistical Analysis

Patient characteristics and comorbidities were summarized as mean and standard deviation (SD) for continuous variables and frequency and proportion for categorical variables. In analysis, age was categorized into three groups: <50, 50–70, and >70 years old. The association with COVID-19 diagnosis was explored utilizing a simple linear logistic regression for each of the potential risk factors and the raw odds ratio (OR) and the 95% confidence interval (95% CI) were calculated for the strength of association. The associations with COVID-19 mortality for the potential risk factors were explored with both simple linear logistic regression for raw ORs and multiple linear logistic regression for adjusted ORs. Potential interactions were evaluated and removed from the multiple logistic regression model if not significant. The sample size of this study was determined by the available and eligible cases from EHR between February 25, 2020 and June 22, 2020 at UAB, including 24,722 COVID-19 negative and 604 COVID-19 positive subjects. This large sample size achieved >80% power to detect even a very small effect (e.g., OR=1.25) in the association of potential risk factors and contracting COVID-19. Among COVID-19 positive subjects 67 were identified as deceased during the study period and this sample size achieved >80% power to detect a medium effect size (e.g., OR=2.4 or smaller depending on the distribution of risk factors) for the association of subject characteristics and mortality. The power analyses were conducted with a two-sided test in a logistic regression under the significance level of 0.05, using PASS 14 Power Analysis and Sample Size Software (NCSS, LLC. Kaysville, Utah). The statistical analyses were conducted using SAS 9.4 (Cary, NC).

## Results

### Subject Characteristics and Coronavirus 2019 Diagnosis

The characteristics of the 24,722 subjects who tested negative for COVID-19 and 604 subjects who had a confirmed positive COVID-19 test are listed in **Table 1**. This low positivity rate of 2.4% is most likely due to the fact that asymptomatic hospital staff and patients coming for elective procedures were included in this screening. To explore the association between COVID-19 diagnosis and potential risk factors, a simple logistic regression was used. Notably, despite only representing 26% of the population in Alabama, the number of African-Americans who tested positive for COVID-19 was disproportionally high as African-Americans represented 52% of those who tested positive while accounting for only 30% of those who tested negative. This resulted in a highly significant odds ratio (OR 2.6, 95% CI 2.19–3.10; p<0.0001) (**Table 1**). In contrast, only 36% of COVID-19 positive subjects were Whites, whereas Whites made up 56% of those who tested negative, further underlining the racial disparity. Interestingly, 70% of all subjects diagnosed with COVID-19 had pre-existing hypertension, 61% had obesity and 40% had diabetes and the risk of being diagnosed with COVID-19 while suffering from any one of these comorbidities was significantly elevated (p<0.0001) (**Table 1**). In the case of diabetes, 92% of subjects also had hypertension and 74% were obese, which may have further contributed to the increased risk observed in this population. Overall, these results are very much in line with global observations and suggested that our cohort provided a representative sample.

**Table 1:** Subject characteristics and COVID-19 diagnosis.

Subject characteristics	Covid-19		Comparison	OR (95%CI)	P-value
	Negative(n = 24,722)	Positive(n = 604)			
Age Group					
<50 years	10626 (43.0%)	239 (39.6%)			
50–70 years	9862 (39.9%)	245 (40.6%)	50–70 vs <50	1.10 (0.92, 1.32)	0.2798
>70 years	4234 (17.1%)	120 (19.9%)	>70 vs 50–70	1.14 (0.91, 1.42)	0.2432
Race					
African-American (AA)	7498 (30.3%)	311 (51.5%)	AA vs White	2.61 (2.19, 3.10)	<0.0001
White	13821 (55.9%)	220 (36.4%)			
Other	3403 (13.8%)	73 (12.1%)			
Sex					
Male	10671 (43.2%)	272 (45.0%)	M vs F	1.06 (0.90, 1.25)	0.4629
Female	13841 (56.0%)	332 (55.0%)			
Unidentified	210 (0.8%)				
Obesity					
Yes	11167 (45.2%)	371 (61.4%)	Y vs N	1.93 (1.64, 2.28)	<0.0001
No	13555 (54.8%)	233 (38.6%)			
Hypertension					
Yes	11891 (48.1%)	420 (69.5%)	Y vs N	2.46 (2.07, 2.93)	<0.0001
No	12831 (51.9%)	184 (30.5%)			
Diabetes					
Yes	5865 (23.7%)	239 (39.6%)	Y vs N	2.11 (1.78, 2.48)	<0.0001
No	18857 (76.3%)	365 (60.4%)			

### Characteristics and Mortality of Coronavirus 2019 Positive Subjects

Overall mortality in COVID-19 positive individuals was 11%, but varied a lot depending on a number of subject characteristics. Ninety three percent of deaths occurred in subjects over the age of 50 and male sex as well as hypertension were associated with a significantly elevated risk of death as assessed by bivariate logistic regression analysis (**Table 2**). In addition, diabetes was associated with a dramatic increase in mortality (OR 3.62; 95% CI 2.11–6.2; p<0.0001). In fact, 67% of deaths occurred in subjects with diabetes.

**Table 2:** Characteristics and mortality of COVID-19 positive subjects.

Subject characteristics	Mortality		Comparison	OR (95%CI)	P-value
	Alive (n = 537)	Deceased (n = 67)			
**Age Group**					
<50 years	234 (43.6%)	5 (7.5%)			
50–70 years	216 (40.2%)	29 (43.3%)	50–70 vs <50	6.28 (2.39, 16.5)	0.0002
>70 years	87 (16.2%)	33 (49.2%)	>70 vs 50–70	2.83 (1.62, 4.93)	0.0003
**Race**					
African-American (AA)	277 (51.6%)	34 (50.8%)	AA vs White	0.84 (0.49, 1.43)	0.5262
White	192 (35.7%)	28 (41.8%)			
Other	68 (12.7%)	5 (7.5%)			
**Sex**					
Male	231 (43.0%)	41 (61.2%)	M vs F	1.52 (1.19, 1.72)	0.0055
Female	306 (57.0%)	26 (38.8%)			
**Obesity**					
Yes	328 (61.1%)	43 (64.2%)	Y vs N	1.14 (0.67, 1.94)	0.6234
No	209 (38.9%)	24 (35.8%)			
**Hypertension**					
Yes	361 (67.2%)	59 (88.1%)	Y vs N	3.60 (1.68, 7.69)	0.001
No	176 (32.8%)	8 (11.9%)			
**Diabetes**					
Yes	194 (36.1%)	45 (67.2%)	Y vs N	3.62 (2.11, 6.20)	<0.0001
No	343 (63.9%)	22 (32.8%)			

**Figure 1:**
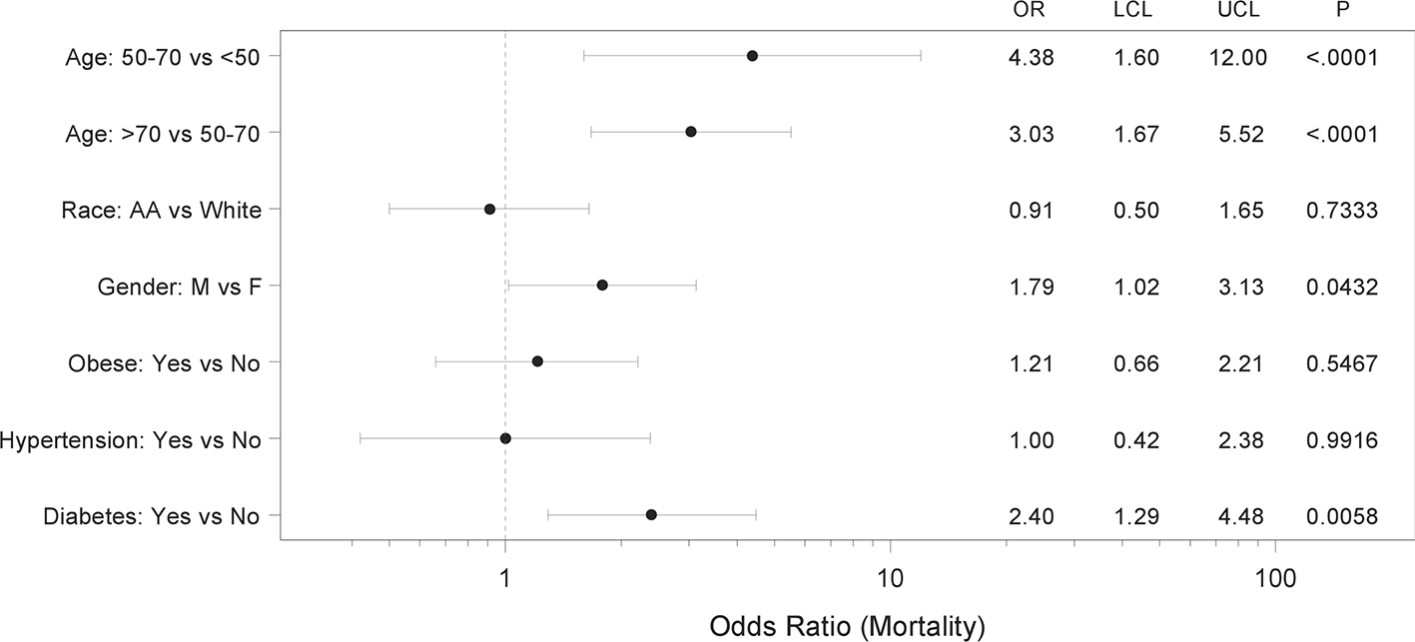
Forest plot showing adjusted mortality risk in subjects with coronavirus 2019 (COVID-19). Multiple logistic regression analysis with age, race, sex, obese status, hypertension status, and diabetes status as covariates was performed. The regression yielded a significant model (p<0.0001) with AUC of 0.79 (95% CI 0.74–0.85) and the adjusted odds ratios (OR), 95% confidence intervals (LCL-UCL) and corresponding P-values are shown.

We also conducted multiple logistic regression analysis with age, race, sex, obese status, hypertension status, and diabetes status as covariates and the adjusted odds ratios and 95% CIs are illustrated in **Figure 1**. Specifically, after controlling for these other covariates, age, sex, and diabetes emerged as the major factors significantly associated with COVID-19 related mortality, suggesting that they are independent risk factors.

### Characteristics and Mortality of Coronavirus 2019 Positive Subjects With Diabetes

Based on the identification of diabetes as an independent risk factor for mortality in COVID-19 positive subjects, we explored potential additional risk factors within this diabetic subgroup. Notably, higher age and male sex continued to be associated with increased mortality in the context of diabetes, while no significant difference between type 1 (T1D) and type 2 diabetes (T2D) was observed (**Table 3**). Next, we investigated the effects of diabetes treatment on adverse COVID-19 outcome. We focused on insulin and metformin as the two most common medications prescribed for T2D. To avoid confounding effects from insulin being initiated for stress hyperglycemia and from metformin being discontinued in hospitalized patients, only medications used prior to the diagnosis of COVID-19 were considered. Interestingly, while prior insulin use did not seem to affect mortality risk, metformin use significantly reduced the odds of dying (OR 0.38; 95% CI 0.17–0.87; p=0.0221). In fact, with 11% the mortality of metformin users was comparable to that of the general COVID-19-positive population and dramatically lower than the 24% mortality observed in subjects with diabetes and not on metformin. Of note, this beneficial effect of metformin use on adverse outcome remained even when subjects with chronic kidney disease or chronic heart failure, classical contraindications for metformin, were excluded from the analysis (OR 0.17; 95% CI 0.04–0.79; p=0.0231). This makes any potential confounding effects from skewing metformin users toward healthier subjects without these additional comorbidities, very unlikely. To further determine whether the effect might be just driven by female sex, as one report proposed that women particularly benefit from metformin (12), we also analyzed males separately. Interestingly, the odds ratio of dying remained significantly lower in male subjects on metformin (OR 0.28; 95% CI 0.09–0.88; p=0.0286).

**Table 3:** Characteristics and mortality of COVID-19 positive subjects with diabetes.

Subject characteristics	Mortality		Comparison	OR (95%CI)	P-value
	Alive (n = 194)	Deceased (n = 45)			
**Age Group**					
<50 years	50 (25.8%)	2 (4.4%)			
50–70 years	104 (53.6%)	20 (44.4%)	50–70 vs <50	4.81 (1.08, 21.4)	0.0392
>70 years	40 (20.6%)	23 (51.1%)	>70 vs 50–70	2.99 (1.48, 6.03)	0.0022
**Race**					
African-American (AA)	127 (65.5%)	28 (62.2%)	AA vs White	0.82 (0.40, 1.68)	0.5855
White	52 (26.8%)	14 (31.1%)			
Other	15 (7.7%)	3 (6.7%)			
**Sex**					
Male	91 (46.9%)	30 (66.7%)	M vs F	2.26 (1.15, 4.47)	0.0187
Female	103 (53.1%)	15 (33.3%)			
**Obesity**					
Yes	144 (74.2%)	34 (75.6%)	Y vs N	1.07 (0.51, 2.28)	0.8539
No	50 (25.8%)	11 (24.4%)			
**Hypertension**					
Yes	176 (90.7%)	43 (95.6%)	Y vs N	2.20 (0.49, 9.84)	0.3027
No	18 (9.3%)	2 (4.4%)			
**Diabetes**					
Type 1 (T1D)	16 (8.2%)	3 (6.7%)	T1D vs T2D	0.79 (0.22, 2.85)	0.7245
Type 2 (T2D)	178 (91.8%)	42 (93.3%)			
**Insulin in T2D**					
Yes	72 (40.5%)	15 (35.7%)	Y vs N	0.82 (0.41, 1.64)	0.5728
No	106 (59.5%)	27 (64.3%)			
**Metformin in T2D**					
Yes	68 (38.2%)	8 (19.1%)	Y vs N	0.38 (0.17, 0.87)	0.0221
No	110 (61.8%)	34 (81.0%)			

**Figure 2:**
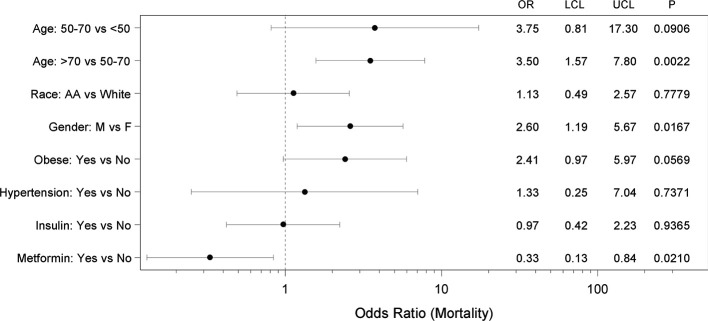
Forest plot showing adjusted mortality risk in subjects with coronavirus 2019 (COVID-19) and T2D. Multiple logistic regression analysis with metformin use, insulin use, age, race, sex, obese status, and hypertension status as covariates was performed and yielded a significant model (p=0.0001) with AUC of 0.77 (0.69, 0.85). The adjusted odds ratios (OR), 95% confidence intervals (LCL-UCL), and corresponding P-values are shown.

Moreover, we again performed multiple logistic regression analysis with metformin use, insulin use, age, race, sex, obese status, and hypertension status as covariates and the adjusted odds ratios and 95% CIs are shown in **Figure 2**. Specifically, after controlling for other covariates, age, sex, and metformin use emerged as independent factors affecting COVID-19 related mortality. Interestingly, even after controlling for all these other covariates, the likelihood of death for subjects taking metformin for their T2D was significantly less than for those who did not take metformin (OR 0.33; 95% CI 0.13–0.84; p=0.0210). In this regard it is also important to note that subjects not taking metformin did not have more severe metabolic disease or diabetes than those on metformin as demonstrated by comparable or even lower body mass index (BMI) and hemoglobin A1C (HbA1C) values (**Table 4**).

**Table 4:** BMI, BG, and HbA1C of subjects with COVID-19 and T2D treated with/without metformin.

		Alive		Deceased		P-value
		Mean	*SD*	Mean	*SD*	t-test
**BMI (kg/m2)**	Metformin use - Yes	35.2	*9.4*	30.9	*6.9*	0.2406
	Metformin use - No	33.6	*8.7*	32.4	*9.8*	0.6039
**HbA1C (%)**	Metformin use - Yes	8.0	*2.6*	7.3	*1.3*	0.4627
	Metformin use - No	7.0	*1.8*	6.6	*2.1*	0.4428
**BG-diagnosis**						
**(mg/dL)**	Metformin use - Yes	181.4	*84.6*	207.3	*81.0*	0.5166
	Metformin use - No	148.6	*58.2*	156.2	*60.2*	0.5799
**BG-illness**						
**(mg/dL)**	Metformin use - Yes	156.6	*48.4*	173.7	*53.9*	0.4713
	Metformin use - No	143.0	*33.1*	147.8	*51.7*	0.6466

Still, since metformin is known and used for its weight neutral or even weight lowering properties, while improving glycemic control in T2D (13), we wondered whether these effects might contribute to the reduced risk of COVID-19 related mortality. However, neither BMI nor HbA1C were lower in metformin users who survived as compared to those who died (**Table 4**). While surprising, this is consistent with the notion that long-term glycemic control does not affect COVID-19 outcome, as recently reported (6). Also, only one subject in our cohort experienced a hyperosmolar hyperglycemic state and ended up surviving and there were no subjects with diabetic ketoacidosis (DKA). Moreover, blood glucose at diagnosis and during the illness were not significantly different in metformin-users who survived as compared to those who died (**Table 4**). This further suggests that other factors may play a more important role in terms of the metformin effects on outcome in the context of COVID-19 and T2D.

## Discussion

In summary, the findings of this study in a racially diverse population demonstrate that diabetes is an independent risk factor associated with increased mortality in individuals with COVID-19, whereas metformin treatment is associated with dramatically reduced mortality in subjects with T2D even after correcting for multiple covariates.

Most strikingly, we found that metformin use prior to the diagnosis of COVID-19 was associated with a ~3-fold decrease in mortality and significantly lower unadjusted and adjusted odds ratios in subjects with diabetes. Of note, this effect remained even after correcting for age, sex, race, obesity, and hypertension or chronic kidney disease and heart failure. Interestingly and in alignment with this finding, an early report from Wuhan, China also suggested that metformin was associated with decreased mortality in hospitalized COVID-19 patients with diabetes in (14). Metformin was also found to be associated with reduced risk of early death in the French CORONADO study (6) and most recently, it was suggested to be associated with decreased mortality in women with COVID-19 based on a UnitedHealth data analysis (12). The fact that such similar results were obtained in different populations from around the world suggests that the observed reduction in mortality risk, associated with metformin use in subjects with T2D and COVID-19, might be generalizable. In fact, a very recent meta-analysis of this collective work concluded that metformin has benefits in reducing the mortality rate from COVID-19 (15). Furthermore, these findings underline the importance of following general diabetes treatment and prevention guidelines and not delaying or discontinuing any metformin treatment. Especially during this pandemic that puts subjects with diabetes at particularly high risk, this treatment might not only help with diabetes management, but also reduce the risk of adverse outcome in case of a COVID-19 infection.

At this point, the mechanisms by which metformin might improve prognosis in the context of COVID-19 are not known. Our findings suggest that they go beyond any expected improvement in glycemic control or obesity as blood glucose, HbA1C, or BMI were not lower in COVID-19 survivors on metformin. Interestingly, metformin has previously been shown to also have anti-inflammatory (16, 17) and anti-thrombotic effects (18, 19) and excessive inflammatory responses, e.g., cytokine storm as well as disseminated thromboembolic events have been recognized as deadly complications of COVID-19 infection (20–22). It is therefore tempting to speculate that by exerting some of its anti-fibrinolytic activities (18) and inhibiting inflammatory cytokines such as tumor necrosis factor alpha or interleukin-6 (16, 17), suspected to play a role in the immune response to COVID-19 (12), metformin might improve outcome. In fact, even prior to the COVID-19 pandemic, preadmission metformin use was found to be associated with reduced mortality in medical and surgical intensive care patients with T2D (23).

While diabetes has been recognized universally as one of the major comorbidities adversely affecting COVID-19 outcome, the factors responsible for this phenomenon are not well understood. Of note, we found that the increased mortality risk of subjects with diabetes persisted even after correcting for covariates such as age, race, obesity, and hypertension, suggesting that while these factors might contribute to a worse outcome, they cannot fully account for it. In the CORONADO study higher glucose levels at admission were associated with a trend toward increased mortality (6) and in-hospital hyperglycemia contributed to worse prognosis in a large multicenter study of patients with COVID-19 from Wuhan (24). Consistently, we found in general slightly higher glucose levels in subjects who died. However, neither blood glucose levels at diagnosis nor during the illness were lower in metformin-users, making it very unlikely that better control of blood glucose was responsible for the improved outcome observed in subjects taking metformin. Also, long-term glycemic control as assessed by HbA1C did not affect mortality in our study, in alignment with previous reports (6). Similar to the issue with metformin, other factors such as diabetes-associated inflammation (25) and coagulopathy (26) may therefore play a more prominent role in this regard. In addition, a recent report also demonstrated that pancreatic beta cells can get infected and damaged by SARS-CoV-2 (27) providing a potential explanation for the extremely high insulin requirements seen in some subject with COVID-19 as well as the development of diabetic ketoacidosis and possibly new onset diabetes (28, 29).

Higher age and male sex were the other independent risk factors associated with increased mortality that we found consistently across subjects with and without diabetes. In fact, the mortality rate in males was more than two-fold higher than in females, which is in line with previous studies (30). Many theories have been proposed for why this might be, including the different concentrations of sex steroids, different fat distribution, different level of circulating pro-inflammatory cytokines, and different innate and adaptive immune response to viral infections (30, 31). In fact, due to this striking sexual dimorphism, studies using anti-androgens in COVID-19 positive men are currently ongoing. In any case, it is encouraging that the beneficial effects of metformin remained strong in the male subjects of our study.

In our cohort being African-American appeared to be primarily a risk factor for contracting COVID-19 rather than for mortality. These findings are supported by a recent study using an integrated-delivery health system cohort with similar demographics (~30% Blacks/African-American), which found that Black race was not associated with higher in-hospital mortality than White race. This suggests that any racial disparity observed may be more likely due to exposure risk and external, socioeconomic factors than to biological differences. The fact that other geographic areas (mostly with a smaller proportion of African-Americans), did see a difference in mortality (10), might be related to issues with healthcare access.

Limitations of the study include the size that did not allow for any separate analyses of additional subgroups such as T1D or subjects on other anti-diabetic drugs besides metformin. On the other hand, the diverse community comprising a large proportion of African-American men and women represents a unique feature of our study. Also, the fact that in our study metformin-users did not have lower blood glucose levels than non-users, suggested that better metabolic control was unlikely to be responsible for the improved outcome observed in these subjects.

Taken together, our study reaffirmed the role of the major comorbidities associated with COVID-19 in a more diverse population with a higher proportion of African-Americans, demonstrated the prominence of diabetes as an independent risk factor associated with higher mortality and revealed that metformin use prior to a diagnosis of COVID-19 was associated with a consistent and robust decrease in mortality in subjects with diabetes. Future studies will have to explore how metformin might confer these protective effects, provide a careful risk benefit assessment and determine whether the indications for metformin treatment should be broadened in the face of the ongoing COVID-19 pandemic.

## Data Availability Statement

The original contributions presented in the study are included in the article/supplementary material. Further inquiries can be directed to the corresponding author.

## Ethics Statement

The studies involving human participants were reviewed and approved by UAB Institutional Review Board. Written informed consent for participation was not required for this study in accordance with the national legislation and the institutional requirements.

## Author Contributions

AC and TG were responsible for data acquisition and analysis. PL performed all the statistical analyses. MM and FO helped with the approach and interpretation. AS conceived the study and wrote the manuscript. All authors contributed to the article and approved the submitted version.

## Conflict of Interest

The authors declare that the research was conducted in the absence of any commercial or financial relationships that could be construed as a potential conflict of interest.
